# Ocular-Motor Profile and Effects of Memantine in a Familial Form of Adult Cerebellar Ataxia with Slow Saccades and Square Wave Saccadic Intrusions

**DOI:** 10.1371/journal.pone.0069522

**Published:** 2013-07-22

**Authors:** Francesca Rosini, Pamela Federighi, Elena Pretegiani, Pietro Piu, R. John Leigh, Alessandro Serra, Antonio Federico, Alessandra Rufa

**Affiliations:** 1 Eye tracking and Visual Application Lab (EVA Lab), Medical, Surgical and Neurological Sciences, University of Siena, Siena, Italy; 2 Veterans Affairs Medical Center, Case Western Reserve University, Cleveland, Ohio, United States of America; 3 Medical, Surgical and Neurological Sciences, University of Siena, Siena, Italy; Barrow Neurological Institute, United States of America

## Abstract

Fixation instability due to saccadic intrusions is a feature of autosomal recessive spinocerebellar ataxias, and includes square wave intrusions (SWI) and macrosaccadic oscillations (MSO). A recent report suggested that the non-competitive antagonist of NMDA receptors, memantine, could decrease MSO and improve fixation in patients with spinocerebellar ataxia with saccadic intrusions (SCASI). We similarly tested two sisters, respectively of 58 and 60 years, with an unrecognized form of recessive, adult-onset cerebellar ataxia, peripheral neuropathy and slow saccades, who showed prominent SWI and also complained with difficulty in reading. We tested horizontal visually guided saccades (10°–18°) and three minutes of steady fixation in each patient and in thirty healthy controls. Both patients showed a significant reduction of peak and mean velocity compared with control subjects. Large SWI interrupting steady fixation were prominent during steady fixation and especially following visually guided saccades. Eye movements were recorded before and during the treatment with memantine, 20 mg/daily for 6 months. The treatment with memantine reduced both the magnitude and frequency of SWI (the former significantly), but did not modified neurological conditions or saccade parameters. Thus, our report suggests that memantine may have some general suppressive effect on saccadic intrusions, including both SWI and MSO, thereby restoring the capacity of reading and visual attention in these and in other recessive forms of ataxia, including Friedreich’s, in which saccadic intrusions are prominent.

## Introduction

Saccadic intrusions occurring during attempted visual fixation are a well-documented feature of recessive hereditary cerebellar ataxias, such as Friedreich’s ataxia and spinocerebellar ataxia with saccadic intrusions (SCASI) [1], [2]. Such movements comprise a range of disorders that include square-wave intrusions (SWI), macrosaccadic oscillations (MSO), saccadic pulses, and ocular flutter [3]. Of these, SWI are the most common and consist of a pair of small, predominantly horizontal saccades (typically <5°), the first of which takes the eye away from the fixation position and the second returns it after a period of 200–400 ms [4]. Since the eye returns to its target following each bi-saccadic intrusion, vision is usually not compromised by square-wave jerks unless they are large and frequent. Many healthy individuals have SWI, and their frequency may be as high as 20 per minute [4]. In hereditary ataxias and parkinsonian disorders such as progressive supranuclear palsy, the size and frequency of SWI may be increased, sometimes being so frequent to appear as “square-wave oscillations.” [3] Recent studies suggest that SWI form a continuum with microsaccades, which all normal subjects display, and which appear to prevent fading of vision due to sensory adaptation [5], [6]. It is hypothesized that, if the size of microsaccades increases, a return movement will be triggered, giving the appearance of SWI [7], [8].

Distinct from SWI are MSO, which consist of a series of predominantly horizontal saccades that oscillate the eyes across the point of fixation [9]. Although MSO also show an intersaccadic interval of 200 ms, they often degrade vision because the line of sight does not land on target, but straddles the point of fixation. MSO often co-exist with saccadic hypermetria, and occur following destructive lesions of the deep cerebellar nuclei [10]. Patients with MSO and saccadic hypermetria often complain of difficulty with reading, which can be explained by their frequently losing their place as they switch from one line of text to the next [3]. Other saccadic intrusions such as saccadic pulses and ocular flutter may also disrupt vision, but they are less common that SWI and MSO in the hereditary ataxias.

A recent report [11] suggested that the non-competitive antagonist of NMDA receptors memantine [12] could decrease MSO and improve vision in patients with hereditary ataxia. We report a similar effect of memantine in two sisters with an unrecognized form of adult-onset cerebellar ataxia and severe axonal neuropathy, who showed prominent SWI.

## Materials and Methods

We studied two sisters, ages 58 and 60 years, who developed during their fourth decade progressive spino-cerebellar ataxia, severe axonal neuropathy slow saccades and SWI that caused them to report difficulty with reading (both were mathematics teachers). A complete panel of clinical, neuro-ophthalmologic, laboratory and neuroimaging assessment was applied in these patients including genetic testing for rare and neuro-metabolic form of ataxias. Visual acuity for distance and near was performed before and during treatment by means of standard eye charts including the Jaeger chart for reading at 30–40 cm of distance. The international cooperative ataxia rating scale (ICARS) [13] was scored before and after treatment. A group of 30 healthy age-matched individuals (range 38–68 years) served as control subjects.

### Ethics Statement

All subjects and patients gave their written informed consent; the patients in this manuscript have given written informed consent (as outlined in PLOS consent form) to publish these case details; the study was approved by the Local Ethic Committee: Comitato Etico Locale Azienda Ospedaliera Universitaria Senese, and performed according to the Declaration of Helsinki.

### Saccade Behaviour and Session Testing

Eye movements were recorded by means of an eye tracker device (ASL 504, Applied Science Laboratories, Bedford, MA, USA). Data acquisition and visual stimulus were controlled by a PC-compatible Pentium 3 GHz computer running a custom software dedicated to real-time data management. Eye position was sampled with a frequency of 240 Hz, digitized with a resolution of 16-bit, corresponding to a sensitivity of recording eye position of 0.16°, and stored for off-line post-processing analysis. The visual stimulus was shown on a LCD screen having a dimension of 51×31 cm and a resolution of 1024×768 pixels at 72 cm of distance. An interactive procedure based on nine static points of calibration and three static points of validation were performed to ensure a minimization of spatial error. The subject's head movements were minimized by a chinrest with a bite bar.

In a series of independent experiments, patients underwent a total of two blocks of visually guided horizontal saccade tests and one block of fixation test during each session of recording. The visual stimulus of both tasks was a red dot (diameter 0.2° of visual angle, luminance 63 cd/m^2^ on a black background (luminance 2.5 cd/m^2^). Horizontal saccades were elicited in responses to target presentations at two possible eccentricities, 10° and 18° of amplitude. After the central fixation point disappeared, a peripheral target appeared for 1500–2000 ms to the right or left eccentricity in an unpredictable manner. Each saccade test consisted in a sequence of 20 or 40 trials depending on the patient’ compliance. Finally, in the fixation test we recorded eye movements during three minutes of attempted steady fixation.

Eye movements were measured before and during the treatment. After the baseline measurements (before any treatment), the two patients started to take memantine in slowly increasing dose from 5 mg/day, up to 20 mg/day at which their eye movements were recorded again. Ocular movements of both patients were recorded on two or more separate days before and during therapy.

### Data Analysis

A post-processing analysis of saccade parameters was conducted off-line using a computer algorithm developed in the Matlab environment (The MathWorks Inc., Natick, MA, USA). Recorded data of eye position were processed to remove blinks, filtered using a low-pass filter (third-order Butterworth low-pass digital filter with 3 dB attenuation at 25 Hz), and differentiated using an eight-point central difference method in order to determine the velocity [14]. The identification of starting and ending times of all saccadic eye movements were performed automatically using an algorithm based on a velocity threshold (velocity threshold >10°/s). Saccades with a latency lesser than 100 ms were considered as spontaneous or anticipatory; and saccades with a direction greater than 30° in respect to the horizontal direction were also rejected automatically. Finally, an interactive method was used to verify the correctness of automatic selection of saccades and to correct it, if necessary. We determined saccadic parameters [15] for each initial saccade defined as the first saccade performed after target presentation. We considered saccade latency, calculated as the time between stimulus presentation and saccade onset; peak and mean velocity, calculated as the maximum eye velocity and the ratio between saccade amplitude to duration, respectively, measured in degrees of visual angle/second; duration, calculated as the time interval between the start and the end of the eye movement; amplitude, calculated as the difference between eye position at the start and end of the saccade, in degrees of visual angle; gain, calculated as the ratio of the initial saccade amplitude to target distance; and accuracy evaluated using the absolute error, calculated as the modulus of the angular distance between target position and final eye position of the initial saccade. Since small saccades (under 8 degrees) could have a similar behaviour to SWI, we selected saccades with magnitude less than 8 degrees and determined variation of the same saccadic parameters before and during treatment in both patients.

The identification and characterization of the SWI was conduct using a interactive semi-automated method based on the automatic identification of saccadic eye movement [14], algorithm described above, combined with visual inspection of both horizontal and vertical eye positions. SWI appeared as two opposite small saccadic with similar amplitude, separated by a short interval. To further characterize their dynamic properties SWI were selected using an amplitude threshold of 8° (saccades have to be less than of 8°), a velocity limit of 580°/sec and an intrasaccadic interval threshold of 500 ms. Finally, the reciprocal amplitude, the direction of the saccade pairs and the duration of the intersaccadic interval were assessed for each SWI. SWI were characterized by the duration of the intersaccadic interval (Inter-SI) and the duration of intrasaccadic interval (Intra-SI); the frequency, the amplitude and peak velocity of both the first and the return saccade. The intersaccadic interval (Inter-SI) was the time interval between each pair of consecutive SWI, from the end of the second saccade of one SWI and the start of the first saccade of subsequent SWI; the intrasaccadic interval (Intra-SI) was the time interval between the end of first saccade and the start of the return saccade constituting a SWI; the frequency was the number of saccadic intrusions measured per minute (n. of SWI/min); the amplitude and the peak velocity were the amplitude, in degrees of visual angle, and the maximum eye velocity in degrees/second of two opposite saccades that constituted an intrusion. We also subdivided the three minutes of fixation in smaller trials using a temporal window of 30 seconds moving at 15 seconds of constant increments. We calculated the mean rate of SWI for each subtrial and compared the values before and after treatment in each patient.

### Statistical Analysis

The Shapiro-Wilk test was used to verify the normality for each variable; Levene’s to test for equality of variances between the couples of variables; the *t* test was used to compare the couples of variables; and the Mann-Whitney was applied when the condition of normality was not met. Post-hoc analysis was performed with the Holm-Sidak test [16]. All the statistical tests were two-tailed and run at the significance level of 5%. The saccadic parameters of both patients before treatment were compared with controls. After testing for inter trial differences among saccade parameters between the two patients, overall saccade parameters were pooled and compared before and during treatment. Total least squares (TLS), i.e. orthogonal regression, was used to study the relationship of the main sequence between the peak velocity and amplitude. The root means square errors (RMSE) were calculated to determine the goodness of fitting of the TLS.

## Results

### Clinical Findings

The clinical findings and ICARS score are summarized in Table 1. Both sisters showed normal visual acuity for distance (logMAR: 0.1), color sensitivity, steroacuity, and fundoscopy. However, they both complained of reduced speed, and frequent loss of the word place, when reading. The best- corrected visual acuity during reading was 3 and 4 Jeager (1.0 and 0.8 M letter size) for Patient 1 and 2 respectively. Both patients were tested negative for a wide range of genetic, toxic and metabolic causes of adult-onset ataxia, including Friedreich’s ataxia, other autosomal recessive forms of cerebellar ataxia and SCASI. Functional parameters and ICARS score were unchanged after one month of treatment with memantine whereas, the best corrected visual acuity during reading improved both subjectively and objectively; it was 2 Jeager (0.5 M letter size) in both sisters.

**Table 1:** Main clinical features of both patients, including ICARS subscores.

Clinical Findings	Patient 1	Patient 2
Sex/age at onset/age at examination	F/30–40 y/58 y	F/30–40y/60 y
Symptoms-signs at onset	Gait in-coordination	Gait in-coordination
Visual functions	Normal	Normal
Trouble with reading	++	++
Dysarthria	+	++
Liquid dysphagia	+	+
Dysmetria	++	+++
Lower limbs hypotonia	+	+
Brisk tendon reflexes	+	+
Gait ataxia	++	+++
EMG Axonal sensory neuropathy	++	+++
MRI global cerebellar atrophy	++	++
ICARS subscoresunchanged after therapy		
*Posture and gait disturbances*	18	25
*Kinetic functions*	17	27
*Speech disorders*	5	5
*Oculomotor disorders*	6	6
*Global score*	46	63

### Saccadic Intrusions

**Figure 1:**
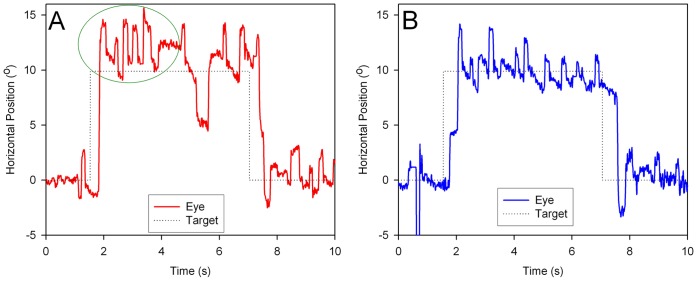
Records of horizontal gaze during a saccade test from Patient 1 before (A) and during (B) treatment. Although saccadic intrusions are present during the periods of fixation on the center target (evident at the beginning and end of the figure), the greatest frequency of intrusions occurs following the rightward saccade at about 2 seconds into the record (highlighted within the ellipse). During treatment with memantine, saccadic intrusions are still present, but tend to be smaller, less frequent and do not increase after the rightward saccade. Positive values indicate rightward eye and target movements.

Figure 1A shows a representative record of saccadic intrusions occurring during a saccade task prior to starting memantine therapy; they mainly conformed to large SWI, with pairs of saccades away from and then back to the fixation point. Although these intrusions occurred during fixation, they were often more prominent after a gaze shift (highlight with oval in Figure 1A). Figure 1B shows the same patient during a similar saccade task while she was taking memantine at a dose of 20 mg/day. Although SWI are still present, they are less frequent, smaller, and do not occur as a flurry of intrusions after the voluntary saccade, as they did prior to treatment (Figure 1A).

Since the SWI variables of amplitude and peak velocity for each patient were not distributed normally (Shapiro-Wilk, p<0.0001), the Mann-Whitney test was applied.

#### Patient 1

Before therapy, the mean frequency of SWI was 93/min (SD±12), mean amplitude 3.3° (SD±1.2), mean peak velocity 185.9°/sec (SD±68.8). With therapy SWI rate reduced to 86/min (SD±40) (p>0.05), while amplitude and peak velocity significantly reduced respectively to mean amplitude 2.9° (SD ±0.9, p<0.001) and mean peak velocity 170.5°/s (SD ±64, p<0.001).

#### Patient 2

**Figure 2:**
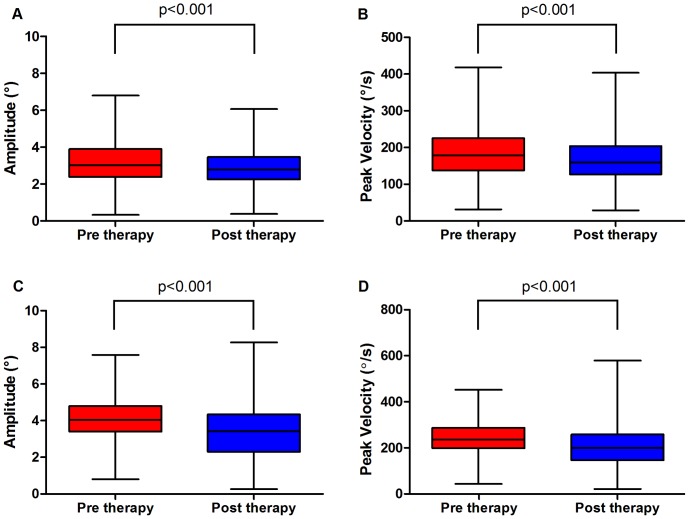
Box-whiskers of amplitude and peak velocity (pre vs post therapy). A–B: Variations of amplitude and peak velocity values of saccadic intrusions of Patient 1. C–D: Variations of amplitude and peak velocity values of Patient 2.

Before therapy the frequency of SWI was 102/min (SD±10), mean amplitude 4.1° (SD±3.3), mean peak velocity 241.0°/sec (SD±74). During treatment, SWI rate decreased to 96/min (SD±44) (p>0.05); whereas the amplitude and peak velocity significantly reduced (mean amplitude 3.4°, SD±1.3, p<0.001; mean peak velocity 209.1°/sec, SD±87, p<0.001). See Figure 2 and Table 2 for a summary.

**Table 2:** Main SWI parameters (data of both patients are showed).

	Patient 1			Patient 2		
SWI parameters	Pre	Post	Significance	Pre	Post	Significance
**InterSI**	(426±373) ms	(653±795) ms	***P = 0.01***	(166±181)ms	(295±229) ms	P>0.05
**IntraSI**	(115±54) ms	(108±60) ms	***P = 0.03***	(123±51) ms	(98±52) ms	***P = 0.001***
**Frequency**	93/min	86/min	P>0.05	102/min	96/min	P>0.05
**Peak velocity**	(185.9±68.8) °/s	(170.5±64) °/s	***P<0.001***	(241.0±74) °/s	(209.1±87) °/s	***P<0.001***
**Amplitude**	(3.3±1.2)°	(2.9±0.9)°	***P<0.001***	(4.1±3.3)°	(3.4±1.3)°	***P<0.001***

The mean Inter-SI of Patient 1 was significantly (p = 0.01) longer after memantine (pre: 426 ms, SD ±373, range: 48–1768 vs post: 653 ms, SD ±795; range 58- 4775). In Patient 2 the mean Inter-SI interval increased but not significantly (p>0.05) between pre and post conditions (pre: 166 ms, SD ±181, range: 4.2–972 vs post: 295 ms, SD ±229,0 range: 8.42–1347).

The Mean Intra-SI showed a significant reduction with therapy in both sisters. Patient1: Intra-SI pre 115 ms (SD±54) and post 108 ms (SD±60) (p = 0.03); Patient 2: Intra-SI pre 123 ms (SD ±51), post 98 (SD ±52) (p = 0.001).

**Figure 3:**
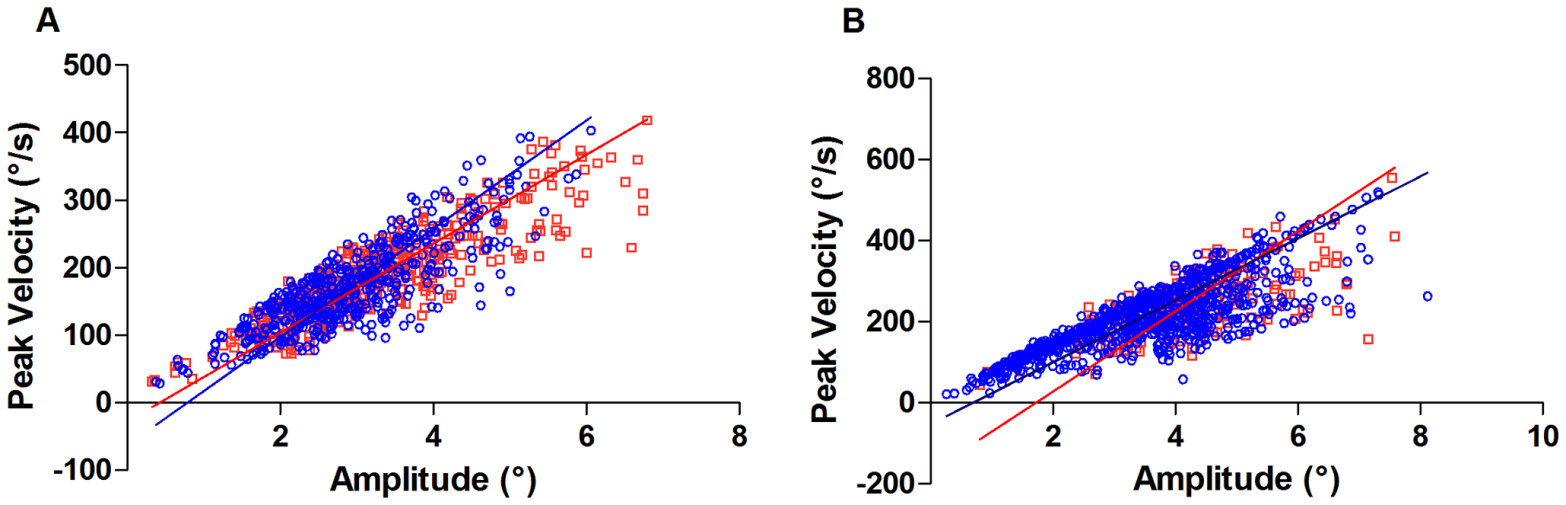
The SWI peak velocity–saccade amplitude relationship (the main sequence) of saccadic intrusions. Data were obtained with TLS regression (see methods). Data points are saccadic intrusions of patients pre-therapy (open red rectangle) and post-therapy (open blue circle). The colored lines are fitted to the respective data. A: Patient (Pt)1. B: Patient (Pt)2. RMSE: Pre Pt1: 0.5873; Post Pt1: 0.5771; Pre Pt2 : 0.8565; Post Pt2: 0.7689. Slope: Pre Pt1: 65.88; Post Pt1: 80.22; Pre Pt2: 99.05; Post Pt2: 76.46. Y-intercept: Pre Pt1: −27.96; Post Pt1: −63.07; Pre Pt2: −170.5; Post Pt2: −53.06.

The variation in amplitude and peak velocity of SWI and the main sequence of peak velocity vs amplitude of the intrusions of both patients before and during treatment are shown respectively in Figure 3. No significant differences were found between the slopes of data plotted before and after treatment in Patient 1 (RMSE pre:0.5873; Slope pre: 65.88; RMSE post:0.5771; Slope post: 80.22; p = 1); in Patient 2, slope of data after treatment was significantly lower than slope of data pre (RMSE pre:0.8565; Slope pre: 99.05; RMSE post:0.7689; Slope post: 76.46, p<0.001).

### Saccades (Table 3)

Since no inter trial differences in saccade parameters were found among patients, the data of both sisters in pre conditions were pooled and compared with those obtained during treatment.

**Figure 4:**
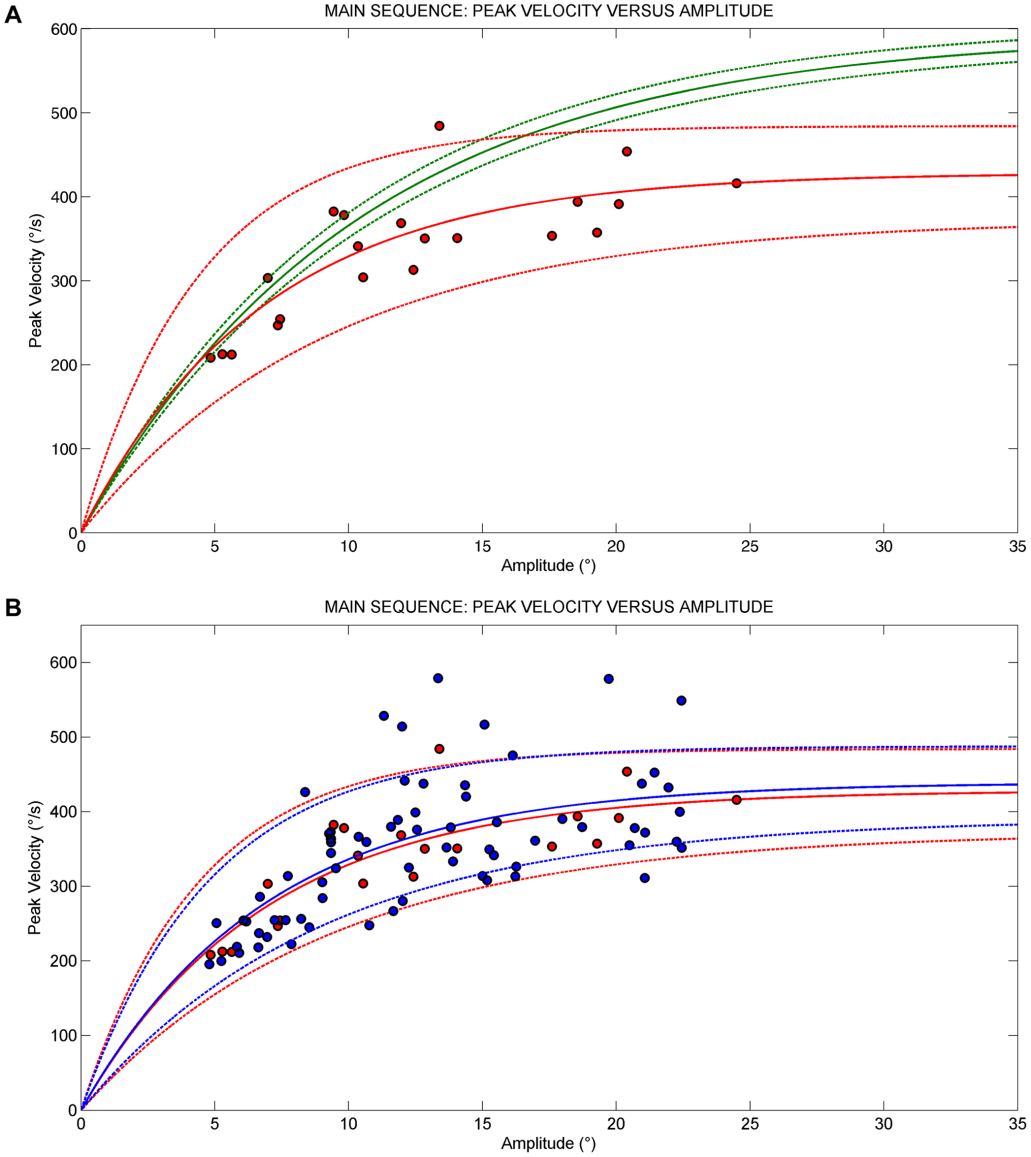
Plot of Main Sequence relationships. A) Red data points are saccades of patients (filled red circle). The red curves were fitted to the respective data; the green curves fitted the controls data (data points not shown). B) Data points are saccades of patients pre-therapy (filled red circle), and patients post-therapy (filled blue circle). The colored curves were fitted to the respective data. The data were fitted using the exponential equation V_peak_ = V_max_ [1−e^(−amplitude/C)^], where V_max_ is asymptotic peak velocity and C is the angle constant shaping the exponential rise. The 95% prediction bounds for healthy subjects are indicated (broken curve).

Table 3 summarizes the main saccadic parameters of patients vs controls. Peak velocity (t-test, p<0.001), mean velocity (t-test, p = 0.02) and accuracy (t-test, p = 0.009) were significantly lower in patients than controls at 10° and particularly at 18° (p<0.001) of target eccentricity. Latencies (t-test, p<0.001) were significantly higher than controls at 10° and 18°. The Peak velocity-amplitude relationship in patients and controls is reported in Figure 4A.

**Table 3:** Main saccade parameters of Patients Vs Controls (significant data shown).

Saccade parameters		Patients	Controls	Statistical significance
**Latency**	**18°** **10°**	(275±72) ms	(182±23) ms	***p<0.001***
		(351±31) ms	(189±17) ms	***p<0.001***
**Amplitude**	**18°** **10°**	(9.2±3.1)°	(10.3±0.62)°	p = 0.21
		(17.0±5.2)°	(18.3±0.7)°	p = 0.5
**Peak velocity**	**18°** **10°**	(300.8±69.4) °/s	(385.4±41.8) °/s	***p<0.001***
		(385.2±60.4) °/s	(513.2±59.2) °/s	***p<0.001***
**Mean velocity**	**18°** **10°**	(164.8±51.2)°/s	(207.2±22.2) °/s	***p = 0.02***
		(207.4±33.1)°/s	(277.1±25.4) °/s	***p<0.001***
**Gain**	**18°** **10°**	(0.89±0.29)	(1.02±0.6)	p = 0.16
		(0.91±0.28)	(0.98±0.04)	p = 0.5
**Accuracy**	**18°** **10°**	(2.5±1.8)°	(0.9±0.3)°	***p = 0.009***
		(3.7±3.7)°	(1.2±0.5)°	p = 0.07
**Duration**	**18°** **10°**	(52±7)ms	(48±4)ms	p = 0.13
		(78±24)ms	(65±4)ms	p = 0.13

No significant differences were found during treatment (t-test p>0.05) for all the saccadic parameters.

Since hypometric saccades with amplitude less than 8 degrees could have dynamic characteristics similar to the SWI and could be erroneously included in the selection of SWI, we extracted separately saccades with amplitude less than 8 degrees and compared their dynamics in pre and post conditions. We found that in the pre condition, the percentage of these small saccades was 28%, with an amplitude ranging from 4.8° to 7.4°; the percentage in the post-condition was 20% with a magnitude ranging from 4.7° to 7.8°. No significant differences were found among all saccadic parameters between the two conditions (t-test p>0.05).

The peak velocity-amplitude relationship pre vs during treatment is reported in Figure 4B.

## Discussion

We report here an unrecognized, probably recessive hereditary form of adult-onset cerebellar ataxia clinically characterized by axonal sensory neuropathy, cerebellar atrophy, slow saccades and prominent SWI.

We set out to determine whether frequent and large saccadic intrusions occurring in two sisters with identical phenotype, could be suppressed with the drug memantine, which is an uncompetitive antagonist of the NMDA-receptor (NMDAR) [12]. Our impetus to carry out this study was partly stimulated by the report that MSO occurring in patients with a recently defined recessive form of spinocerebellar ataxia (SCASI) were suppressed with memantine, leading to improved vision [11]. Our patients’ saccadic intrusions conformed more to SWI than to MSO, since they did not appear to oscillate the eyes around the fixation point. Nonetheless, our patients’ saccadic intrusions did cause visual symptoms, possibly because of their size and because they were prominent after gaze shifts (Figure 1A), such as would occur during reading. Moreover our patients’ saccades were slow with normal-to-hypometric amplitude compared with saccades in SCASI, which are hypermetric and hyper-fast. Thus, SCASI patients showed MSO associated with saccade hypermetria suggesting a prevalent cerebellar midline dysfunction [11], which was dissimilar from our patients. This would indicate a different form of familial adult onset cerebellar ataxia in our two sisters. The patients described here showed an identical phenotype characterized by severe spino-cerebellar ataxia, diffuse axonal neuropathy, global cerebellar atrophy, slow saccades, and quite similar saccadic intrusions, including their responses to memantine. Notably, memantine did not improve global motor performance (ICARS remained unchanged), or saccade dynamics (including small saccades), but significantly reduced the amplitude, and to a lesser extent, the frequency of SWIs in each patient. Although the reduction of the frequency of SWI with memantine was not statistically significant, the inter-saccadic interval, an indirect measure of SWI frequency, was longer during memantine therapy particularly in the younger sister. We also noted declines in the speed of SWI with memantine therapy, but these may be largely due to reduced amplitude.

Unlike MSO, which cross the midline (fixation point) and severely affecting reading, SWIs shift the eye around the fixation point and do not cross the midline; therefore they are thought to have no visual consequences. However, as is evident in Figure 1, SWI were more prominent and larger immediately following saccadic gaze shifts, and it seems possible that they would interfere with the patient’s ability to move from one line of text to the next.

Thus, our report suggests that memantine may have some general suppressive effect on saccadic intrusions, including both SWI and MSO. Recent studies [17] suggest that SWI are part of a spectrum with normally occurring microsaccades, the return saccade being generated when the size of the primary movement takes the image of the feature of interest off the foveal area of the retina [5], [7]. MSO would appear to be a form of hypermetria of square-wave intrusions, in which each saccade overshoots the point of fixation because of a general cerebellar saccadic hypermetria. Since memantine appears to be effective for both SWI and MSO, then its predominant action would seem to be due to a common effect on the generative mechanism for these intrusive saccades rather than on the cerebellar mechanism causing hypermetria. If this is the case, memantine might prove effective, to some extent in all forms of “square-wave oscillations”, including SWI and MSO [18]–[20]. Whatever the exact mechanism or mechanisms in this complex circuit, the current and prior reports suggest the need to conduct a controlled trial of memantine, preferably a cross-over study with placebo, to treat saccadic intrusions, and their effects on the quality and efficiency of vision, in patients with recessive ataxia, of which the most common is Friedreich’s [1].
